# Evaluation of the Changes of the Intercanine and Intermolar Widths Following Palatal Expansion in the Mixed Dentition Patients with Bilateral Posterior Crossbite: A Systematic Review

**DOI:** 10.3390/dj11020052

**Published:** 2023-02-13

**Authors:** Yen Nie Lim, Fadzlinda Baharin, Galvin Sim Siang Lin, Rozita Hassan, Milton Hongli Tsai, Lim Chia Wei, Suzanne Yeoh, Mark Ko Xiang Ping

**Affiliations:** 1 Paediatric Dentistry Unit, School of Dental Sciences, Universiti Sains Malaysia, Health Campus, Kubang Kerian 16150, Malaysia; 2 Department of Dental Materials, Faculty of Dentistry, Asian Institute of Medicine, Science and Technology (AIMST) University, Bedong 08100, Malaysia; 3 Orthodontic Unit, School of Dental Sciences, Universiti Sains Malaysia, Health Campus, Kubang Kerian 16150, Malaysia; 4 Discipline of Orthodontics, Department of Family Oral Health, Faculty of Dentistry, Universiti Kebangsaan Malaysia (UKM), Kuala Lumpur 50300, Malaysia; 5 Taman Intan Dental Clinic, Ministry of Health Malaysia, Sungai Petani 08000, Malaysia; 6 Tudan Dental Clinic, Tudan Health Clinic, Ministry of Health Malaysia, Miri 98000, Malaysia; 7 RTC Sibuti Dental Clinic, Bangunan RTC Sibuti, Ministry of Health Malaysia, Bekenu 98150, Malaysia

**Keywords:** orthodontics, malocclusion, crossbites, mixed dentition, palatal expansion technique, systematic review

## Abstract

This systematic review aimed to identify the intercanine and intermolar width changes following palatal expansion in bilateral posterior crossbite (PXB) in mixed dentition. This review was registered in the PROSPERO database (CRD42021275833). All randomized controlled trials (RCT) and non-RCT articles between 1980 and August 2022 on the palatal expansion of bilateral PXB in mixed dentition were searched in seven online databases (Google Scholar, Ovid, Web of Science, Scopus, EBSCOHost, Cochrane Library and PubMed). The risk of bias (RoB) of the articles included was analyzed using the Joanna Briggs Institute (JBI) critical appraisal tool. Three non-RCT studies were included and showed a low risk of bias. Meta-analysis on the changes in intercanine and intermolar widths was not performed due to study design heterogeneity. One study reported an over-correction of the bilateral PXB. There is a need for more RCT studies with standardized landmark measurements, outcome assessment methods and retention periods to investigate the interdental changes following palatal expansion.

## 1. Introduction

The definition of posterior crossbite (PXB) is an inadequate transverse relationship between maxillary and mandibular teeth where the buccal cusps of maxillary teeth occlude with the central fossae of the mandibular teeth [1]. The prevalence of PXB in children ranges between 8–20% [2,3]. The possible etiologies of PXB are a non-nutritive sucking habit and mouth breathing, adenotonsillar hypertrophy or chronic allergy [4,5,6,7,8,9]. Furthermore, PXB is associated with temporomandibular joint disorder, which includes muscular pain, headache and clicking [10]. In children, uncorrected PXB will result in reduced bite force in chewing or clenching, less active masseter muscle on the crossbite side compared with the non-crossbite side and more active anterior temporalis muscle [11]. 

Early correction of PXB aims to achieve a better tooth/skeletal relationship, develop a symmetrical condylar position with glenoid fossa and correct the asymmetrical muscular activity [10,12]. Posterior crossbite is not self-corrected if no intervention is done, and it should be managed as early as possible [13,14,15,16]. Early crossbite correction can be carried out with a coffin spring, quad helix appliance, rapid maxillary expansion (RME), NiTi expanders and fixed orthodontic appliances [17]. Maxillary expansion is achieved by separating the mid-palatal sutures or dentoalveolar movement [18]. As cited in Kutin and Hawes [16], Wertz [19] recommends differentiating between unilateral and bilateral PXB. A true, unilateral PXB where the midlines coincide should be managed unilaterally. A unilateral crossbite that arises from lateral mandibular displacement is commonly caused by bilateral maxillary constriction. A bilateral PXB may have resulted from a severe bilateral constriction. In mild bilateral disharmony, a lateral mandibular displacement may arise due to tooth interference, which results in unilateral PXB. In this case, the unilateral PXB should be managed using bimaxillary expansion. 

The decision in orthodontics treatment planning between maxillary expansion and extraction management has been debated over the years [20]. Suggested palatal expansion for a patient with maxillary deficiency to “broaden the smile” while gaining space after transverse palatal expansion was the point of interest for the clinician in treatment planning [21,22,23,24,25,26,27]. For orthodontic treatment planning, it is essential to predict arch width, perimeter, and arch length changes after palatal expansion [22,28]. 

Apart from space gains, changes in soft tissue from extraction should be considered during orthodontic treatment planning. Rapid maxillary expansion in growing children will significantly increase the width of the nose, mouth, upper philtrum, and the distance from the lower lip to the E-line [29,30]. By contrast, orthodontic treatment with extraction in Class I malocclusion resulted in more retracted upper and lower lips than non-extraction orthodontic treatment [31]. In cases of extraction of premolars, about 12 per cent of the patients presented with flattened facial profiles after the completion of orthodontic treatment [32].

A systematic review and meta-analysis on the effectiveness of early orthodontic correction of unilateral PXB [33] reported that Quad Helix (QH) was more effective than expansion plates (EP) in increasing the intermolar width and reducing the treatment time. This study also showed that the relapse rate was greater in the QH group than in the EP group at 5.6 years post-treatment. The study on the interdental changes following palatal expansion in mixed-dentition children with bilateral PXB was limited. Hence, this systematic review aimed to determine the intercanine and intermolar changes following non-surgical palatal expansion in children with bilateral PXB. 

## 2. Materials and Methods

The present systematic review was conducted following the PRISMA (Preferred Reporting Items for Systematic Reviews and Meta-Analyses) Statement guidelines and the Cochrane Handbook for Systematic Reviews of Interventions [34]. The research question was formulated according to PICO format (Population, Intervention, Comparison and Outcome): what are the intercanine and intermolar width changes following non-surgical expansion in mixed dentition children with bilateral PXB? The PICO criteria were: (1). Population: mixed dentition children with bilateral PXB (2). Intervention: non-surgical palatal expansion (3). Comparison: control group (untreated subjects) (4). Outcome: intercanine and intermolar width.

### 2.1. Review Registration

The current study has been registered in the PROSPERO database (CRD42021275833).

### 2.2. Study Design and Eligibility Criteria

In the present study, the inclusion criteria were children with mixed dentition or at the age of 6 to 13 years old, non-surgical palatal expansion techniques, clinical studies (prospective or retrospective) and the articles being written in English. Studies on surgical palatal expansion, Class III malocclusion with protraction therapy only, cleft lip and palate or another craniofacial syndrome diagnosis, obstructive sleep apnea condition; case report and case series, review articles and expert opinions were excluded. 

### 2.3. Search Strategy

The electronic search was performed using seven online databases: MEDLINE (via PubMed), The Cochrane Library, Web of Science, Ovid, Google Scholar, EBSCOHost and Elsevier (via Scopus). A search of all the RCT and non-RCT articles was performed between 1980 and August 2022. The filter was set to include articles written in English only. The search strategy consisted of terms such as the following: “palatal expansion”, “palatal expansions technique”, “maxillary expansion”, “mixed dentition”, “children”, “bilateral posterior crossbite” and “malocclusion”. Hence, the below search strategy was applied: [(palat* expan*) OR (palatal expansions technique) OR (maxillary expansion) AND (mixed dentition*) OR (child*) AND (bilateral crossbite) OR (bilateral posterior crossbite) OR (malocclusion)].

### 2.4. Selection of Studies

According to the eligibility criteria for inclusion or exclusion, the titles and abstracts of studies were retrieved from the databases and screened independently by four investigators (M.H.T., L.C.W., S.Y., M.K.X.P.). The same four team members retrieved the full text of the potentially eligible studies and independently assessed them for eligibility. When necessary, the authors of the study were contacted for further clarification. Discussion with the fifth (F.B.) investigator resolved any disagreement between them regarding the eligibility of the studies.

### 2.5. Data Extraction and Data Analysis

A standardized and pre-piloted data extraction form was used to extract data from the studies included, for evidence synthesis. Two investigators (F.B., G.S.S.L.) extracted data independently. When necessary, discrepancies were identified and resolved through discussion with another investigator (R.H.), an expert in the field.

The primary outcome of the search was the changes in the intercanine and intermolar width, and the secondary outcome was the correction of bilateral PXB. The data synthesized from the search was put into table form.

### 2.6. Risk-of-Bias Assessment

Methodological quality (risk-of-bias) assessment of the final studies included was carried out using Joanna Briggs Institute (JBI) critical appraisal tools based on the type of study [35]. Two investigators (F.B., G.S.S.L.) independently performed the risk-of bias assessment. Any disagreement was resolved with the third investigator (R.H.). Risk-of-bias assessment was carried out according to the type of study design. 

## 3. Results

### 3.1. Result of the Search

**Figure 1:**
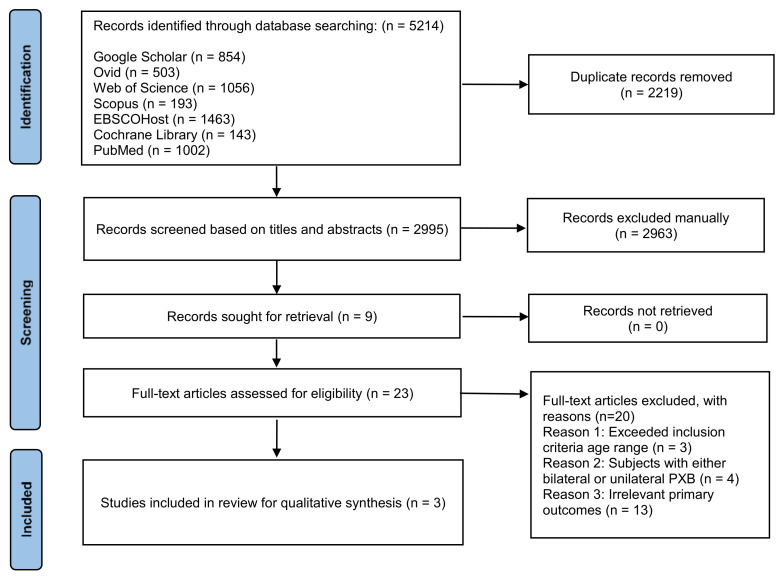
Study selection based on PRISMA guidelines.

As illustrated in Figure 1, from the initial search, 5214 articles were identified. Deduplication removed 2219 records and eliminated 2963 after screening of the title content and abstract. The full-text assessment was carried out on 23 articles in compliance with the inclusion and exclusion criteria. Three studies that complied with the inclusion criteria were included in this study. The main reasons for exclusion were as follows:

- 3 exceeded inclusion criteria age range
- 4 involved subjects with either bilateral or unilateral crossbite
- 13 primary outcomes were irrelevant to our study

### 3.2. Study Characteristics

The characteristics of the studies included were tabulated in Table 1, and the outcomes were tabulated in Table 2. 

#### 3.2.1. Characteristics of Setting

**Location**

Of the three included studies, one study was from Turkey [36], one study was from Brazil [37], and one study was from Spain [38]. 

**Type of Study**

All the studies included were non-RCTs, and all the studies were prospective cohort studies [36,37,38].

**Sample size**

The included studies had 10 [37], 14 [36] and 15 [38] subjects, respectively.

#### 3.2.2. Characteristics of Participants

**Age**

The range of age of subjects was from 7 to 13 years old. The subjects of one study [36] had a mean age of 12.8 ± 1.02 years old. Another study [37] had the mean age of the subjects 8.3 ± 1.24 years old. The mean age of one study was 9.78 ± 2.4 years old [38].

**Dentition stage**

Two studies mentioned that the subjects were in mixed dentition [37,38]. One study did not mention the dentition stage of subjects [36]. 

#### 3.2.3. Characteristics of Interventions and Comparisons

**Type of expansion and appliances used**

The RME technique was used in all three studies [36,37,38]. One study used an acrylic-bonded appliance in palatal expansion [36], while another used a Hyrax appliance [38]. A Haas appliance was used in another study [37]. 

**Comparison group**

One study compared RME with the control group of untreated subjects [37]. Another study compared RME with the compression-to-decompensation technique [38]. One study did not have a comparison group [36]. 

**The method of outcome assessment**

One study [38] used a dental cast to assess intercanine and intermolar width changes. Both posterior-anterior film and dental cast were used to assess the outcome in one study [36]. Another study used a lateral cephalogram and dental cast to assess the outcome [37]. 

**Retention period**

Two studies showed six months retention period [36,37]. Only one study showed a retention period of 3-6 months [38].

#### 3.2.4. Outcomes

**Resolution of bilateral PXB**

One study showed over-corrected bilateral PXB [37]. Two studies did not mention the resolution of bilateral PXB [36,38]. 

**Changes in the intercanine width**

The changes in the intercanine width after palatal expansion were 3.8mm [38], 3.42mm [36] and 3.21mm [37], respectively. 

**Changes in the intermolar width**

The changes in the intermolar width after palatal expansion were 6.2mm [38], 5.42mm [36] and 3.21mm [37], respectively. 

### 3.3. Risk of Bias

All three studies were considered to have a low risk of bias according to the JBI Critical Appraisal Checklist for Cohort Studies (Table 3).

## 4. Discussion

From this review, the included studies [36,38] showed that intermolar width expansion was greater than intercanine width expansion, similar to the other studies [39,40,41]. The possible reason could be that the teeth were moved by the RME appliance attached to the molars and the canines were unattached, in addition to buccal crown tipping [39]. However, this finding contradicts some studies that revealed greater transverse expansion at the anterior region than at the posterior region [42,43]. It was found that the separation of maxillary palatine was non-parallel or wedged-shaped, with a wider opening at the anterior region [44,45,46]. In Wertz’s study [47], one adult and two mixed dentition dry skulls revealed that the separation of the palate was anteroposteriorly non-parallel. For clinical management, to ensure the palatal expansion has taken place, upper occlusal radiography should be taken to visualize the separation of the mid-palatal suture. If there is no sign of separation, it is advised that the activation should be stopped to prevent alveolar fracture or periodontal damage [48].

Another possible factor of greater increase in intermolar width could be attributed to the facial type of the subjects. Rozzi [49] studied the dentoskeletal effects of RME in early mixed dentition with different facial types. According to the study, the high-angle patients (hyperdivergent subjects) showed a greater increase in the buccal tipping of the molars and the intermolar width than the normal- and low-angle subjects (hypodivergent subjects). The increased tipping of the molars and intermolar width probably occurred as the hyperdivergent subjects had a weaker masseter muscle strength and hence lower dental anchorage that requires greater buccal tipping. In contrast, the hypodivergent subjects’ masticatory muscles were stronger, allowing greater masticatory force and stability of the anchor teeth that had a similar function to the bite block [50]. As the dental anchorage intended for torque maintenance was greater in hypodivergent subjects, the palatal expansion was more uniform, and greater intercanine width was ensured [49]. The normal- and low-angle subjects’ intercanine width was more stable than the hyperdivergent subjects. It was evident that higher and more consistent maxillary expansion, fewer dental effects with RME and more permanent long-term outcomes were attainable in normal and hypodivergent patients. This study showed that the palatal expansion had a greater dental effect in high-angle subjects, especially at the molars. In the short term, the torque increased and recurred in the long term. The increased torque on the anchoring molars caused less anterior expansion (intercanine width) and similar intermolar width to other facial-type groups but with greater buccal tipping of the molars [49]. This study [49] advised the usage of the bite-block in hyperdivergent subjects during the palatal expansion as it promoted the anti-rotation of the mandible and allowed greater dental anchorage by preventing excessive buccal tipping of the molars. 

All the studies included [36,37,38] employed RME as the technique for non-surgical palatal expansion. In RME, heavy and rapid forces were applied to the posterior teeth, and there was insufficient time for the teeth to move in response to the force. The forces were transferred to the sutures instead. When the appliance delivered forces that exceeded the capacity needed for orthodontic tooth movement and sutural resistance, the sutures separated, while the teeth moved minimally. The appliance gradually opened the mid-palatal suture and all other maxillary sutures while compressing the periodontal ligament, bending the alveolar process and tipping the anchor teeth. When the maxillary molars were already buccally inclined to compensate for the transverse skeletal discrepancy and the transverse discrepancy was equal to or more than 4 mm, RME was indicated. RME was used in the maxillary protraction of class III malocclusion, moderate maxillary crowding, cleft lip and palate patients and lateral discrepancies that result in posterior crossbites [48,51]. The advantage of using RME was that the orthopaedic changes were greater in RME than in slow maxillary expansion (SME) [52]. The studies suggested that the RME techniques enhance skeletal displacements while minimizing lateral tooth movements [45,53].

Overcorrection in PXB was mentioned in one study [37], where the palatal expansion stopped when the palatal cusp of the upper first molar touched the buccal cusp of the lower first molar. Notably, the other two studies [36,38] did not mention the resolution of PXB, so we assumed all the PXB cases were corrected. Some authors recommended overcorrection of PXB to compensate for the uprighting of the buccal tipping teeth after the retention period, commonly seen in tooth-borne expanders [14,54,55]. It was found that molars return to their original buccolingual inclination after retention stops. If overcorrection were performed, there would be no relapse of PXB [55]. An overcorrection of 2-3 mm during activation would give rise to occlusal interference when the palatal cusps of the maxillary teeth occlude against the buccal cusp of the mandibular teeth, which contributed to increased vertical dimension [49]. In contrast, a study by Petrén et al. [56] showed that the results of palatal expansion were stable without overcorrection of PXB in mixed dentition. The appliance was adjusted for buccal root torque to prevent buccal tipping of molar roots.

The age range of the subjects in the included studies was from 7 years old to 13 years old, with or without mention of the mixed dentition stage. The age of the subjects included was similar to the study of Šlaj et al. [57]. This study investigated the dental arch changes of children with mixed dentition for two years. Two analyses on the dental arch changes were performed, with the first at the early mixed dentition (8-12 years old) and the second at the late mixed dentition (10-14 years old). The eruption of certain permanent teeth defines the dentition stage. No significant difference was yielded from the dental arch changes in the two-year period. Hence, the study concluded that the time between the early and late mixed dentition was appropriate for the environmental influences to interfere with the pattern of dental arch development.

In comparison, a study [13] investigated the treatment timing for RME by evaluating the differences in dentoskeletal changes of RME before and after pubertal growth. The dentition stage of the subjects was not considered in this study. The results reported that when RME was performed in subjects before the pubertal peak, there were significant changes at the skeletal level while the expansion after the pubertal growth spurt showed more expansion at the dentoalveolar level. This finding was explained in the review article of Bell [46], that the formation of mechanical interlocking at maxillary sutures increased the resistance of skeletal expansion as the patient’s age increased. 

No statistical analysis was performed in this review as the studies included showed study design heterogeneity. However, the intercanine and intermolar width changes could aid in the decision of expansion with or without extraction in patients with mixed dentition and bilateral PXB. Only post-retention measurements were considered to estimate the space gain for orthodontic treatment planning. According to Ricketts [21], every 1mm increase in the intermolar distance adds 0.25mm to the perimeter, a ratio of 1:1 for intercanine distance. Perimeter change may be determined approximately by using the equation of the addition of 0.54 times the intercanine expansion and 0.87 times the arch length [58]. However, the study did not mention the presence of bilateral PXB in the subjects.

The study design heterogeneity of this review was contributed by the varied retention period. Two studies [36,37] with a retention period of six months showed no significant relapse and stable expansion after two years and three years of follow-up, respectively. The finding of this review was similar to the finding of another systematic review [59], which concluded that six months of retention of crossbite correction used 24 hours a day may prevent relapse and ensure minimal changes in short-duration follow-up. The duration of retention contributed to the stability of the palatal expansion. The study of Mew [54] suggested that the measurement of relapse be taken two to four months after the retention period, as it appeared to be more stable. This review paper proposed future studies to standardize the retention period and measurement of the stability of the palatal expansion in PXB. 

Another factor of the study design heterogeneity was that different landmarks were used to measure the intermolar width. The landmarks used to measure the intermolar width were the distance between the midpoint of the buccal and palatal cusp, the distance between the mesiobuccal cusp tip, the distance between mesiopalatal cusp tip, and the union between the palatal gingival margin and tooth. The standardization of landmarks in measuring transverse dental changes was strongly suggested to reduce the heterogeneity. In addition, the measurement instrument used was widely varied, including a gauge calliper, digital calliper and 2-point compass. Only one study [37] analyzed measurement error and coefficient reliability. The potential experimental errors were due to the observer’s fatigue, inconsistency of landmark, less precise measuring instrument and worn-off dental cusp tips, which could be overcome by digitalizing the dental cast. One present study [60] evaluated the skeletal and dental relationships of Haas and Hyrax for RME from dental casts and anteroposterior cephalograms using the 3D technique. Laser scanner and computer software programs were employed to obtain data in three coordinates. This methodology was proven to be accurate, less time-consuming and inexpensive once the scanning protocol had been established. 

Another possible contribution of heterogeneity was the variety of appliances used in the studies included. Banded expanders were Hyrax [38] and Haas [37] expanders, while bonded [36] were acrylic expanders. Banded expanders placed the bands on the maxillary first premolars and molars. Two acrylic pads were in close contact with the palatal mucosa, while a midline jackscrew was incorporated into it, a Haas-type expander. A midline jackscrew located in the palate with proximity to the palatal profile was called a Hyrax-type expander. Commonly using acrylic material with a midline jackscrew, a bonded expander had full coverage of the occlusal surface and partial coverage of the buccal and palatal surfaces of the teeth. Bonded expander’s retention relied on the close-fitting nature of acrylic material to the tooth surface with luting cement. A Haas expander, a tissue-tooth-born banded expander, produced a more parallel expansion as the force on the teeth and the alveolar processes were more evenly distributed [53,61]. The finding of one study [37] was similar in that intercanine and intermolar width differences were the same due to the parallel palatal expansion when using the Haas expander. However, this finding was contradicted by another study that used a Haas expander [43]. The study showed that the midpalatal suture opened in a parallel direction with great buccal tipping on the maxillary first permanent molars. Another study [62] reported a similar result: the banded expander showed more buccal tipping of the first molar and premolar than the bonded expander. 

All the studies included were from three continents [36,37,38], which cannot fully represent the continental population. As the dentition stage was not mentioned, the children with the presence of canines were implied as the indicator of mixed dentition. However, this could be inaccurate as the canines present in both mixed and permanent dentition. In future studies, the correction of PXB should be defined clearly regarding the position of the upper molar’s palatal cusp and the lower molar’s buccal cusp [37]. There is no standardized landmark in measuring the intermolar width, which could cause variation in palatal expansion measurement. Using the gingival margin as the measurement landmark could reduce the error that may have resulted from the buccal tipping of the crown during palatal expansion [22]. The limitation of the review process is the omission of literature not written in English, which may result in language bias. Some of the articles were not retriable, which could have led to reporting bias due to missing papers.

The changes in the intercanine and intermolar widths following palatal expansion might increase the arch circumference, thereby reducing the discrepancy between the tooth size and the arch length. Hence, palatal expansion should be considered when there is buccal crowding. However, when the anterior crowding is observed, a three-dimensional appliance should be incorporated to enhance the intercanine width changes. The finding of this review supports the current theory and practice of growth modification in growing children, especially in correcting the transverse discrepancy in class III malocclusion.

## 5. Conclusions

Our review showed that there are changes in the intercanine and intermolar widths following palatal expansion in mixed dentition children with bilateral PXB. No statistical analysis was performed on the interdental width post-expansion due to the study design heterogeneity. The limitation in methodology of included studies suggests that future RCT studies with the standardized landmark of measurement, method of outcome assessment, and retention period are required to evaluate the changes in interdental width among mixed dentition children with bilateral PXB children.

## Figures and Tables

**Table 1:** Characteristics of included studies.

Study	Country	Type of Study	Sample Size	Mean Age	Dentition Stage	Expansion Type	Appliances Used	Comparison Group	Outcome Assessment	Retention Period
Memikoglu et al. [36]	Turkey	Prospective, cohort study without a control group	14	12.8 ± 1.02	Not mentioned	RME	Acrylic bonded appliance	N/A	Posterior-anterior film Dental cast	6 mths
Micheletti et al. [37]	Brazil	Prospective cohort study with a control group	10	8.3 ± 1.24	Mixed dentition	RME	Haas appliance	Control group (untreated)	Lateral cephalogram Dental cast	6 mths
Lorente et al. [38]	Spain	Prospective cohort study without a control	15	9.78 ± 2.4	Mixed dentition	RME	Hyrax appliance	Compression to decompensation technique	Dental cast	3–6 mths

Abbreviation: RME, rapid maxillary expansion; N/A, not applicable.

**Table 2:** Correction of bilateral PXB and the changes in the intercanine and intermolar width after palatal expansion.

Study	Correction of Bilateral PXB	Intercanine Width (mm)	Intermolar Width (mm)
Memikoglu et al. [36]	Not mentioned	Cusp tip: 3.42	Cusp tip: 5.42
Micheletti et al. [37]	Over-correction	Cusp tip: 3.21 (0.4)	Cusp: 3.21 (0.8)
Lorente et al. [38]	Not mentioned	Cusp tip: 3.8 (2.2)	Cusp tip: 6.2 (2.9)

Abbreviation: PXB, posterior crossbite.

**Table 3:** JBI risk-of-bias quality assessment for cohort studies.

Study	Q1 ^a^	Q2	Q3	Q4	Q5	Q6	Q7	Q8	Q9	Q10	Q11	Percentage of Yes	Risk ^b^
Memikoglu et al. [36]	Yes	N/A	Yes	Yes	Yes	Yes	Yes	Yes	Yes	Yes	Yes	100%	Low
Micheletti et al. [37]	Yes	Yes	Yes	Unclear	Unclear	Yes	Yes	Yes	Yes	N/A	Yes	80%	Low
Lorente et al. [38]	Yes	Yes	Yes	Yes	Yes	Yes	Yes	Yes	N/A	Yes	Yes	100%	Low

Abbreviation: N/A, not applicable.^a^ Q1-Q11 indicate JBI risk-of-bias assessment questions 1 to 11. ^b^ Risk of bias was ranked as high when the percentage of “yes” was ≤ 49%, moderate when the percentage of “yes” was from 50% to 69%, and low when the study percentage of “yes” was ≥ 70%.

## Data Availability

Not applicable.
